# Supplementary material for: A Nonsense Variant in the DMD Gene Causes X-Linked Muscular Dystrophy in the Maine Coon Cat
Source: Animals (Basel). 2022 Oct 25;12(21):2928. doi: 10.3390/ani12212928 (PMC9653713; doi:10.3390/ani12212928)
Supplement: Supplementary file 1 [file animals-12-02928-s001.zip › animals-1951834-supplementary.pdf]

Supplementary information

1. Tables

Table S1. Monoclonal antibodies used for indirect immunofluorescence. NCL-SPEC2 was used as a control for membrane integrity and NCL-MHCd to detect regenerating fibers.

Name	Target	Dilution used	Source
NCL-DYS1	Dystrophin rod domain	1:100	Novocastra Laboratories, UK
NCL-DYS2	Dystrophin carboxy terminus	1:100	Novocastra Laboratories, UK
NCL-DRP2	Utrophin	1:20	Novocastra Laboratories, UK
a-SG	α-sarcoglycan	1:200	Gift of Eva Engvall [48]
NCL-βSG	β- sarcoglycan	1:100	Novocastra Laboratories, UK
NCL-γ-SG	γ-sarcoglycan	1:100	Novocastra Laboratories, UK
L-α2	Laminin α2	1:200	Gift of Eva Engvall [48]
NCL-SPEC2	Spectrin	1:10	Novocastra Laboratories, UK
NCL-MHCd	Myosin heavy chain	1:20	Novocastra Laboratories, UK

Table S2. mRNA expression – Primers. Primer information, amplicon length, and amplicon location based on transcript variant X8 (Acc. No. XM_045050794.1).

Primer name	Primer sequence	Amplicon length	Amplicon location
cDNA-F1:	5'-GGGAAGCAGCACATAGAGAACCT-3'	231 bp	Exons 3-5
cDNA-R1:	5'-ACCAAGAGTCAGTTTGTGATTTCCATC-3'		
cDNA-F2:	5'-GAACAGATGGTGAATGAGGGTGTT-3'	217 bp	Exons 19-20
cDNA-R2:	5'-ATGTGGTGGTAGGCTGGGTT-3'		
cDNA-F3:	5'-TGCCATCTTCCTTGCTGTTGG-3'	228 bp	Exons 51-52
cDNA-R3:	5'-AATTCTGGGCGGCGGTAATG-3'		
cDNA-F4:	5'-TCTGATGACGCAGCCCTGTT-3'	444 bp	Exons 56-58
cDNA-R4:	5'-TTCCCACTCGGTGTTGACCT-3'		
cDNA-F5:	5'-TCAGCTTATAGGACCGCCATGAA-3'	225 bp	Exons 64-65
cDNA-R5:	5'-ACACATATCCACGCAGAGAGGG-3'		

Table S3. mRNA expression – PCR/sequencing information. PCR/sequencing mixes and programs used for each primer pair. Primer pair numbering is based on Table S2.

Primer pair	PCR mix used	PCR program used
PPcDNA-1	1	1
PPcDNA-2	3	2
PPcDNA-3	1	2
PPcDNA-4	2	2
PPcDNA-5	1	2

**PCR mix 1:**<br>5.7 µl H_2O (Thermo Fisher Scientific, Waltham, MA, USA)<br>1.0 µl 10x Key buffer (VWR International, Radnor, PA, USA)<br>1.0 µl Primers (5 µM each) (Integrated DNA Technologies, Coralville, IA, USA)<br>0.2 µl dNTPs (10 mM each) (VWR International)<br>0.1 µl TEMPase Hotstart DNA polymerase (5 U/µl) (VWR International)<br>2.0 µl Template<br>10.0 µl Total volume	**PCR program 1:**<br>14'30" - 95°C<br>00'30" - 95°C]<br>00'30" - 63°C] x 35<br>01'00" - 72°C]<br>04'00" - 72°C<br>Hold - 15°C
**PCR mix 2:**<br>4.7 µl H_2O<br>1.0 µl 10x Key buffer<br>1.0 µl Primers (5 µM each)<br>1.0 µl GC-rich (Roche Diagnostics, Switzerland)<br>0.2 µl dNTPs (10 mM each)<br>0.1 µl TEMPase Hotstart DNA polymerase (5 U/µl)<br>2.0 µl Template<br>10.0 µl Total volume	**PCR program 2:**<br>14'30" - 95°C<br>00'30" - 95°C]<br>00'30" - 61°C] x 35<br>01'00" - 72°C]<br>04'00" - 72°C<br>Hold - 15°C
**PCR mix 3:**<br>3.7 µl H_2O<br>1.0 µl 10x Key buffer<br>1.0 µl Primers (5 µM each)<br>2.0 µl GC-rich<br>0.2 µl dNTPs (10 mM each)<br>0.1 µl TEMPase Hotstart DNA polymerase (5 U/µl)<br>2.0 µl Template<br>10.0 µl Total volume	
**Sequencing mix:**<br>3.0 µl H_2O<br>2.0 µl 5x SEQ-buffer (Thermo Fisher Scientific, Waltham, MA, USA)<br>1.5 µl Sequencing primer (2 µM)<br>1.0 µl GC-rich<br>0.5 µl RR-mix (Thermo Fisher Scientific, Waltham, MA, USA)<br>2.0 µl Template<br>10.0 µl Total volume	**Sequencing program:**<br>2'00" - 95°C<br>0'20" - 95°C]<br>0'10" - 60°C] 30x<br>4'00" - 65°C]<br>Hold - 15°C

Table S4. Transcript variant X8 – primers. Primer information and amplicon length for sequencing of the X8 transcript variant.

Primer name	Primer sequence	Amplicon length
x8-F1	5'-TCACTCACTTTCCCCTTACAGGACT-3'	953 bp
x8-R1	5'-GGATGGCTTCAATGCTCACTTGTT-3'	
x8-F2	5'-GTGGTTTGCCAGCAGTCAGCC-3'	928 bp
x8-R2	5'-GCACCTTATGTTGTTGTACTTGGCGT-3'	
x8-F3	5'-TGGGAAGTCAACTGATTGGAACAGGG-3'	924 bp
x8-R3	5'-TTTGGGGAGGTGGTGGTGGC-3'	
x8-F4	5'-AGCGTGGCTGGACAACTTTGC-3'	915 bp
x8-R4	5'-TCCTGATAGCGCATGGGTGGC-3'	
x8-F5	5'-ACCGGCTTTCAGTTCTTCAGCC-3'	896 bp
x8-R5	5'-AGCTTGCGTCATCCATTCGTGC-3'	
x8-F6	5'-TCAGACAATTCAGCCCAGTCTCAACA-3'	793 bp
x8-R6	5'-TTGCCTCCTCACAGCCTCTTCA-3'	
x8-F7	5'-GAAGGCAAACAAGTGGCTAAACGAAGT-3'	904 bp
x8-R7	5'-TCGCATCTTACGGGACAATTTCAAGCA-3'	
x8-F8	5'-CCTGCGTTGGAAACAAAGAGCGT-3'	775 bp
x8-R8	5'-TTGCCATCAGGTTTGCTGCTTGG-3'	
x8-F9	5'-AGCTGTCACTTCCCGAGCAGA-3'	739 bp
x8-R9	5'-TCTGGGTTCAGGTAGGCTGGC-3'	
x8-F10	5'-TCACAGATGAGAGAAAGCGAGAGGAAA-3'	957 bp
x8-R10	5'-TTTGCCGCTGCCCGATGC-3'	
x8-F11	5'-TGCAGGAAGCTCTCTCACGGC-3'	797 bp
x8-R11	5'-AGGAGCCAGCCACAGAAGCAG-3'	
x8-F12	5'-CGGAGGAACAGTGCTTGTAAGTGC-3'	973 bp
x8-R12	5'-CTCCAGCTTGGCTCTGGCCT-3'	
x8-F13	5'-ACTCATTACCGCCGCCCAGAA-3'	927 bp
x8-R13	5'-AAATCGCCTCCCATCGGTGC-3'	
x8-F14	5'-GGAAGGTTCTGATGACGCAGCC-3'	865 bp
x8-R14	5'-GGAAAGGAAGTGCTGGGACGC-3'	
x8-F15	5'-TGACCTTGCTCGCCAACTCACA-3'	915 bp
x8-R15	5'-ACTCGGTGCAGGACAGGCAG-3'	
x8-F16	5'-GCAAGTGGCAAGCTCCACAGG-3'	817 bp
x8-R16	5'-GGCAGGACTACGAGGCTGGC-3'	
x8-F17	5'-GATTCTGCGCCCGCCTCGT-3'	711 bp
x8-R17	5'-CCCTGTGCTTGTGTCCTGGGG-3'	
x8-F18	5'-GCCGAGGCCAAGGTGAATGG-3'	908 bp
x8-R18	5'-GCCAGCGATTCCAAAAAGAGCAC-3'	
x8-F19	5'-TGGAACGCATTTTGGGTTGTTTA-3'	914 bp
x8-R19	5'-GGCGGAGGGAATTCGTTGTA-3'	
x8-F20	5'-ACTGTGTCCGAGTGATCCGTTAGC-3'	899 bp
x8-R20	5'-ACAAGTGGGTGAAAGCATGTGTGC-3'	
gDNA-F1	5'-AGGGCTTTGCCATGCTCGCAG-3'	425 bp
gDNA-R1	5'-ACAACACTCGTCTACTTCTTCCCACCA-3'	
gDNA-F2	5'-TTAACTTTTGGGGAGTGGGTTTTG-3'	621 bp
gDNA-R2	5'-AAACAATGACAGAACATGCCACA-3'	
gDNA-F3	5'-CTTCTGTGGAAGGCGAGGTAGG-3'	500 bp
gDNA-R3	5'-AGCTACACGTTCTAGGAGCAAACT-3'	

Table S5. Transcript variant X8 – PCR/sequencing information. PCR/sequencing mixes and programs used for each primer pair. Primer pair numbering is based on Table S4.

Primer pair	PCR mix used	PCR program used
PPx8-1	1	2
PPx8-2	1	1
PPx8-3	1	1
PPx8-4	1	1
PPx8-5	1	1
PPx8-6	1	1
PPx8-7	1	1
PPx8-8	1	1
PPx8-9	1	1
PPx8-10	1	1
PPx8-11	1	1
PPx8-12	1	1
PPx8-13	1	1
PPx8-14	1	1
PPx8-15	1	1
PPx8-16	1	1
PPx8-17	1	1
PPx8-18	2	1
PPx8-19	1	2
PPx8-20	1	1
PPgDNA-1	1	1
PPgDNA-2	1	2
PPgDNA-3	1	1

**PCR mix 1:**<br>5.7 µl H_2O<br>1.0 µl 10x Key buffer<br>1.0 µl Primers (5 µM each)<br>0.2 µl dNTPs (10 mM each)<br>0.1 µl TEMPase Hotstart DNA polymerase (5 U/µl)<br>2.0 µl Template<br>10.0 µl Total volume	**PCR program 1:**<br>14'30" - 95°C<br>00'30" - 95°C]<br>00'30" - 62°C] x 35<br>01'00" - 72°C]<br>04'00" - 72°C<br>Hold - 15°C
**PCR mix 2:**<br>4.7 µl H_2O<br>1.0 µl 10x Key buffer<br>1.0 µl Primers (5 µM each)<br>1.0 µl GC-rich<br>0.2 µl dNTPs (10 mM each)<br>0.1 µl TEMPase Hotstart DNA polymerase (5 U/µl)<br>2.0 µl Template<br>10.0 µl Total volume	**PCR program 2:**<br>4'30" - 95°C<br>00'45" - 95°C]<br>00'45" - 66°C] x 35<br>01'30" - 72°C]<br>04'00" - 72°C<br>Hold - 15°C
**Sequencing mix:**<br>3.0 µl H_2O<br>2.0 µl 5x SEQ-buffer<br>1.5 µl Sequencing primer (2 µM)<br>1.0 µl GC-rich<br>0.5 µl RR-mix<br>2.0 µl Template<br>10.0 µl Total volume	**Sequencing program:**<br>2'00" - 95°C<br>0'20" - 95°C]<br>0'10" - 60°C] 30x<br>4'00" - 65°C]<br>Hold - 15°C

Table S6. Primer, probe, and qPCR information for *MYBPC3* genotyping. Sequences of the primers and probes used for *MYBPC3* A31P genotyping are shown, with the resulting amplicon length and the used qPCR mix and program. F and R indicate forward and reverse primers, and Wt and Vt indicate the probes binding to the wildtype and variant strand, respectively.

**Primers**	**Probes**	**Amplicon length (bp)**
F: 5′- GAAGCCAAGGTCAGTG -3′	Wt: FAM-TGTCTCGGCCTCGAA-BHQ1	131
R: 5′- CTAGGCCATACTTGTCAC -3′	Vt: TR- TGTCTCGGGCTCGAA -BHQ2	

**qPCR mix:**	**qPCR program:**
4.9 µl H_2O	14'40" - 95°C
1.0 µl 10x Key buffer	00'20" - 95°C]
1.0 µl Primers (5 µM each)	00'40" - 60°C] x 40
0.4 µl Wt probe (10 µM) (Integrated DNA Technologies)	FAM/TR detection]
0.4 µl Vt probe (10 µM) (Integrated DNA Technologies)	
0.2 µl dNTPs (10 mM each)	
0.1 µl TEMPase Hotstart DNA polymerase (5 U/µl)	
2.0 µl Template	
10.0 µl Total volume	

Figures

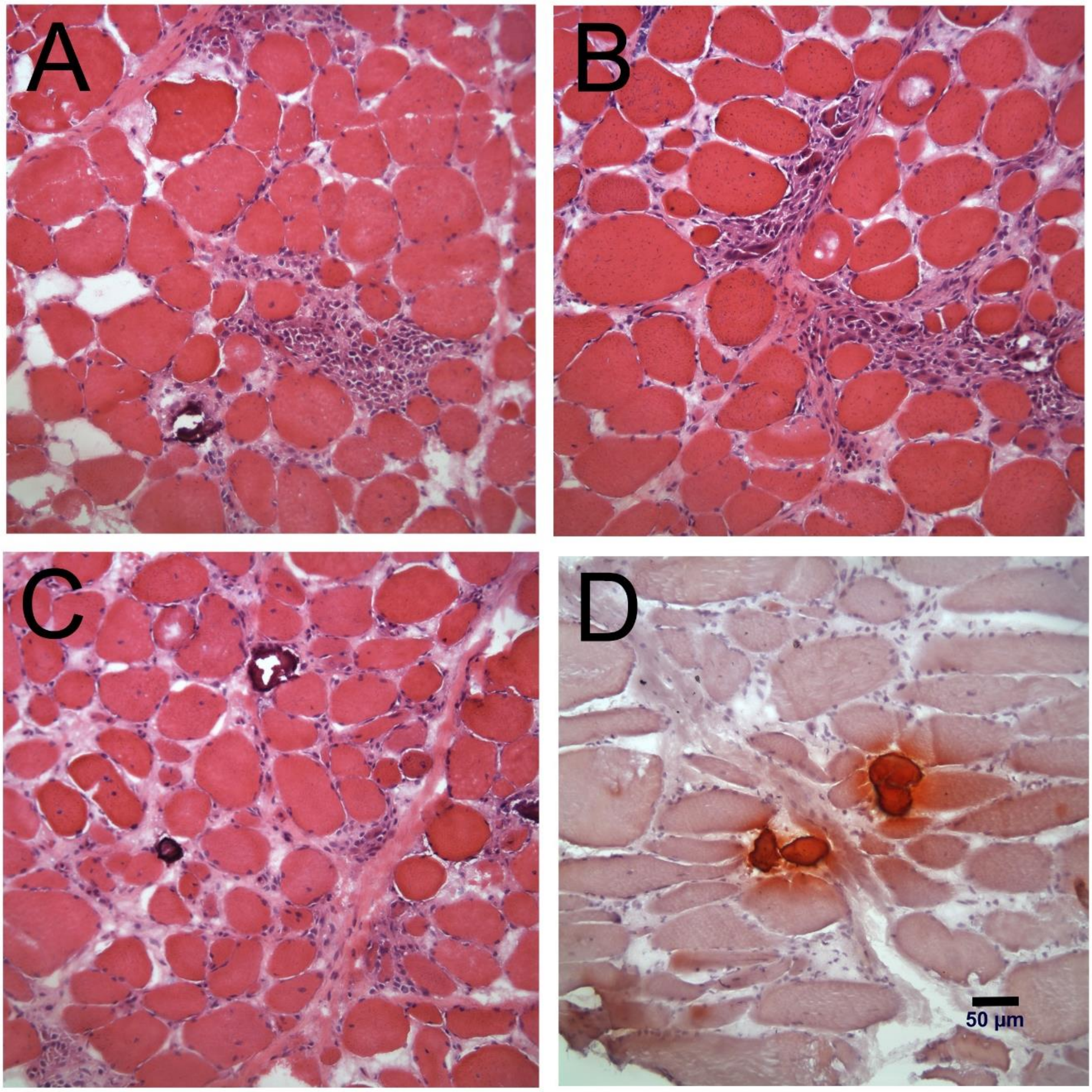

Figure S1. Histopathology of muscle biopsies. Histopathology of muscle biopsies from a Maine coon cat with a myopathic presentation and markedly elevated CK activity (case 1). A) Cryosection from the triceps muscle showing clusters of degenerating fibers. H&E stain. B) Cryosection from the triceps muscle showing clusters of regenerating fibers. H&E stain. C) Cryosection from the vastus lateralis muscle showing scattered calcific deposits. H&E stain. D) Alizarin stain of the vastus lateralis muscle confirming the calcium deposits. Magnification bar in D = 50 µm for all images.

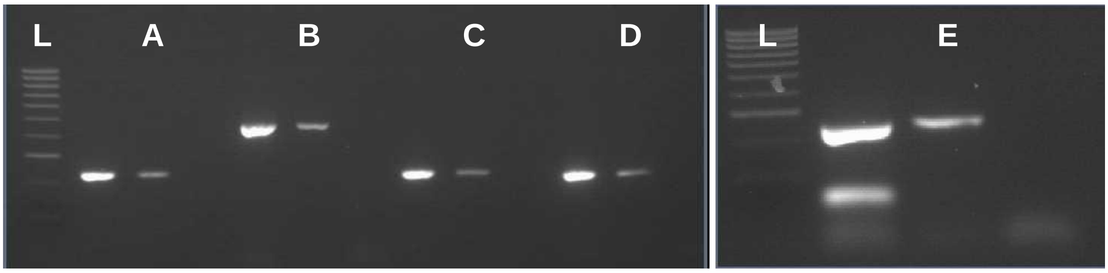

Figure S2. mRNA gel electrophoresis results. Gel electrophoresis results of the 5 PCR reactions inspecting the presence of mRNA in the affected muscle (case 2). L) HyperLadder 100bp (Bioline, UK). A-E) For each primer pair (PP), 10 µl and 2 µl and a non-template control are loaded. Amplicons for PPcDNA-1 (A), PPcDNA-2 (B), PPcDNA-3 (C), PPcDNA-4 (D), and PPcDNA-5 (E) are displayed.
